# Human recreation impacts seasonal activity and occupancy of American black bears (*Ursus americanus*) across the anthropogenic-wildland interface

**DOI:** 10.1038/s41598-022-15665-x

**Published:** 2022-07-16

**Authors:** Tru Hubbard, Michael V. Cove, Diana J. R. Lafferty

**Affiliations:** 1 Wildlife Ecology and Conservation Science Lab, Department of Biology, Northern Michigan University, Marquette, MI 49855 USA; 2 Mammalogy Unit, North Carolina Museum of Natural Sciences, Raleigh, NC 27601 USA

**Keywords:** Behavioural ecology, Conservation biology, Urban ecology

## Abstract

Protected areas serve an important role in wildlife conservation, yet most wildlife occur outside these areas, subject to varying degrees of human disturbance. In the Upper Peninsula of Michigan, American black bears (*Ursus americanus*), a highly mobile, opportunistic species, are common despite an extensive outdoor recreation industry with the potential to affect black bear spatial and temporal activity. We investigated how environmental and anthropogenic factors influence black bear occupancy, detection, and diel activity patterns across the anthropogenic–wildland interface before and after hibernation. Using 30 camera traps deployed across a rural–wildland interface, we captured black bears at 23 camera sites (~ 77%), which exhibited co-occurrence with humans at 10 sites (~ 33%), revealing that human presence and human population density exert negative effects on black bear seasonal occupancy. Bears were more nocturnal during the hunting season, before hibernation. Human recreational activity increased ~ 38% after hibernation, but bear diurnal activity also increased ~ 36%, except when cubs were present. Our results suggest bears prioritize avoiding humans spatially, rather than temporally, except during the hunting season and when cubs are present. Understanding black bear responses to human recreation patterns and environmental variation is essential for minimizing human-mediated disturbance, and fueling conservation efforts of large, charismatic carnivores.

## Introduction

Large mammalian carnivores are often elusive, wide-ranging species that have a history of conflict and controversy surrounding their conservation due to mixed human perceptions^1,2^ and contentious decision-making regarding management, resulting in most carnivore species experiencing continued rapid population declines and loss of habitat worldwide^3,4^. As anthropogenic development continues to increase, carnivores’ large home range size, low population densities, high metabolic demands associated with large body size^4^, and direct persecution due to hunting^5–9^ make them especially vulnerable to landscape changes. Yet, there are still many causes for conservation optimism due to the successful recolonization of some large carnivores across extensive swaths of their historic ranges despite substantial human-modified changes to the global landscape^1,10,11^. Thus, the importance of effective land management and planning that balances the needs of humans and wildlife^2,12^ is critical for promoting effective global carnivore conservation and recolonization^10^.

As urban environments continue to expand, growing evidence suggests that human activity results in a dynamic landscape of fear^13^, in which wildlife, particularly carnivore species with a history of persecution by humans, perceive humans as ‘super predators’^7,14,15^ and respond by modifying their habitat use and behavior^7^. Changes in predator–prey interactions^7^, shifts in diel activity patterns^15–17^ and wildlife movement^18^ associated with human activities have led to increased sightings, nuisance reports, and even increased harvest reports of some species, as well as an unprecedented rise in reported interactions between humans and carnivores^19^. In particular, increased outdoor human recreation, which has become a popular incentive for nature-based tourism and conservation of natural ecosystems, has the potential to cause high levels of ecosystem disruption that may impact carnivore populations and lead to the deterioration of biodiversity^5,20^. For example, as prey species become habituated to human activities associated with nature-based tourism, prey responses to predation risk are rapidly reduced, thus individuals could become vulnerable to other predators in areas where humans are predominant^21,22^. Observing the impacts of human recreation is challenging because wildlife responses to recreation activities can be subtle and vary depending on species, while tracking human presence across the landscape can be unpredictable and difficult to monitor in wild areas^20^. Studies have been done to better understand how recreationists use the landscape of terrestrial wildland and protected areas through the GPS tracking of visitors, which can provide insight for implications on wildlife and improved trail management^23,24^. Urbanization, human population growth, and recreational opportunities are driving people farther into areas where carnivore populations persist, making carnivore behavioral plasticity an important trait for carnivore-human coexistence across landscapes increasingly impacted by humans^9^.

The recolonization of many carnivores in North America is a result of improved management practices supporting landscape connectivity (e.g., corridors)^3^ as well as species becoming more tolerant of developed areas and human activity^3,10,25^, allowing them to persist in human-dominated landscapes and even exploit human resources^10,17^. For example, in urban environments the American black bear (*Ursus americanus*), an omnivorous carnivore, is capable of modifying their foraging behavior^25^ to consume human subsidies such as garbage, fruit trees, and birdseed^26–28^. Although black bears have been recolonizing their former range and even dispersing into new environments (i.e., urban landscapes) over the past several decades^10,19,29^, anthropogenic attractants can lead to more bear-human conflicts. Indeed, American black bears are the most abundant large carnivore in the world^4^, utilizing an array of land cover types (i.e., forest, shrubland, wetland), as well as occupying exurban areas that exhibit lower housing densities and slower development^12,25^. In the state of Michigan (USA), the American black bear population is increasing and expanding farther south in the Lower Peninsula^30^, presenting challenges for wildlife managers, and a growing indifferent public opinion of the species^30–32^.

Following the implementation of successful management practices, black bears are once again recolonizing portions of Mississippi, eastern Texas, Oklahoma^33^, Missouri^3^, portions of urban Connecticut, and farther north into New York^34^ and Maine. In addition, black bear reintroductions in the southeastern U.S. in Arkansas and Louisiana^3^ have also shown to be a successful conservation strategy for the species. Variation in land use among these regions (i.e., forest, agriculture, housing density) and differences in wildlife management policies (i.e., hunting season vs. no hunting season) can have a significant effect on the success of recolonizing populations^12,25^. As such, understanding the influence human activity exerts on the spatial and temporal dynamics of black bears is critical to determine successful management practices of growing carnivore populations that persist across human-dominated landscapes.

For the American black bear, the period of hyperphagia when bears consume excessive food to gain weight as they enter the period of inactivity known as hibernation, plays a key role in their life history and is susceptible to influence caused by changing patterns of human activity, seasonal food availability, and climate^27^. For example, increasing temperatures and expanding urbanization have been postulated to reduce the time of hibernation, further increasing the number of bear-human conflicts along the urban-wildland interface^27^. Increased presence of human features on the landscape (e.g., roads) has resulted in bears increasing nocturnal activity, thus as humans drift farther into remote wild areas, particularly during annual bear hunting seasons, larger circadian shifts in black bear temporal activity may be expected^8,9,35^. Human hunters acting as top predators are restricted in their predation to specific areas and times of the day and year in which hunting is allowed, thus black bears may respond with spatial and temporal variation^8^. For example, during hunting season black bears have been observed to increase their mean distance from non-paved roads, which are highly traversed by hunters during hunting season, while decreasing their distance from paved roads, despite the associated vehicle collision risk^8^. In the Upper Peninsula of Michigan (U.P.), recreational bear hunting (2019 U.P. Bear Hunting Season: September 11–October 26) began in 1925 and has become a long-standing tradition to manage bear populations, but in 2012 license quotas dropped significantly due to expressed concerns from Department of Natural Resources (DNR) biologists and bear hunting clubs^36^. Given this, the investigation of black bear spatial and temporal behavior during the period of “activity” in the U.P. may offer tangible evidence for understanding how recolonizing black bear populations use multi-use lands and respond to anthropogenic activity, which is essential for conservation of black bears in areas where they have not previously persisted and as they recolonize portions of their historic range.

To better understand the influence of human activity on the seasonal spatial and temporal patterns of the American black bear, we used camera traps to examine anthropogenic and environmental factors that have the potential to influence black bear activity and occupancy across the urban-wildland interface of Marquette, MI. The U.P. is home to most of Michigan’s black bears and hosts a growing population that has increased by about 16% since 2012 (i.e., ~ 9902 bears^36^). The pairing of a growing black bear population and popular outdoor recreation scene that hosts a range of activities throughout the year makes the Marquette urban-wildland interface an ideal ecological model system for evaluating black bear behavior relative to human recreation patterns on a seasonal scale. Thus, our research objectives were to determine whether black bears exhibit significant differences in spatial and temporal activity patterns before and after hibernation, while also considering the variation in human activity throughout the year^37^, determine which types of human activity and environmental factors influence black bear detection and occupancy across the landscape, and determine if black bears display a shift in their activity patterns following the hunting season. We predicted that black bear activity would be driven by the energy demands of hyperphagia before hibernation causing black bears to occupy a greater proportion of the landscape in early fall, prior to their decrease in activity to retain fat stores in late fall. Similarly, the need for larger quantities of food and nutrients for cubs will increase black bear activity level following hibernation. Finally, we predicted that black bears would exhibit higher nocturnality in the fall due to the increased risk associated with direct persecution via hunting.

## Materials and methods

### Study area

**Figure 1:**
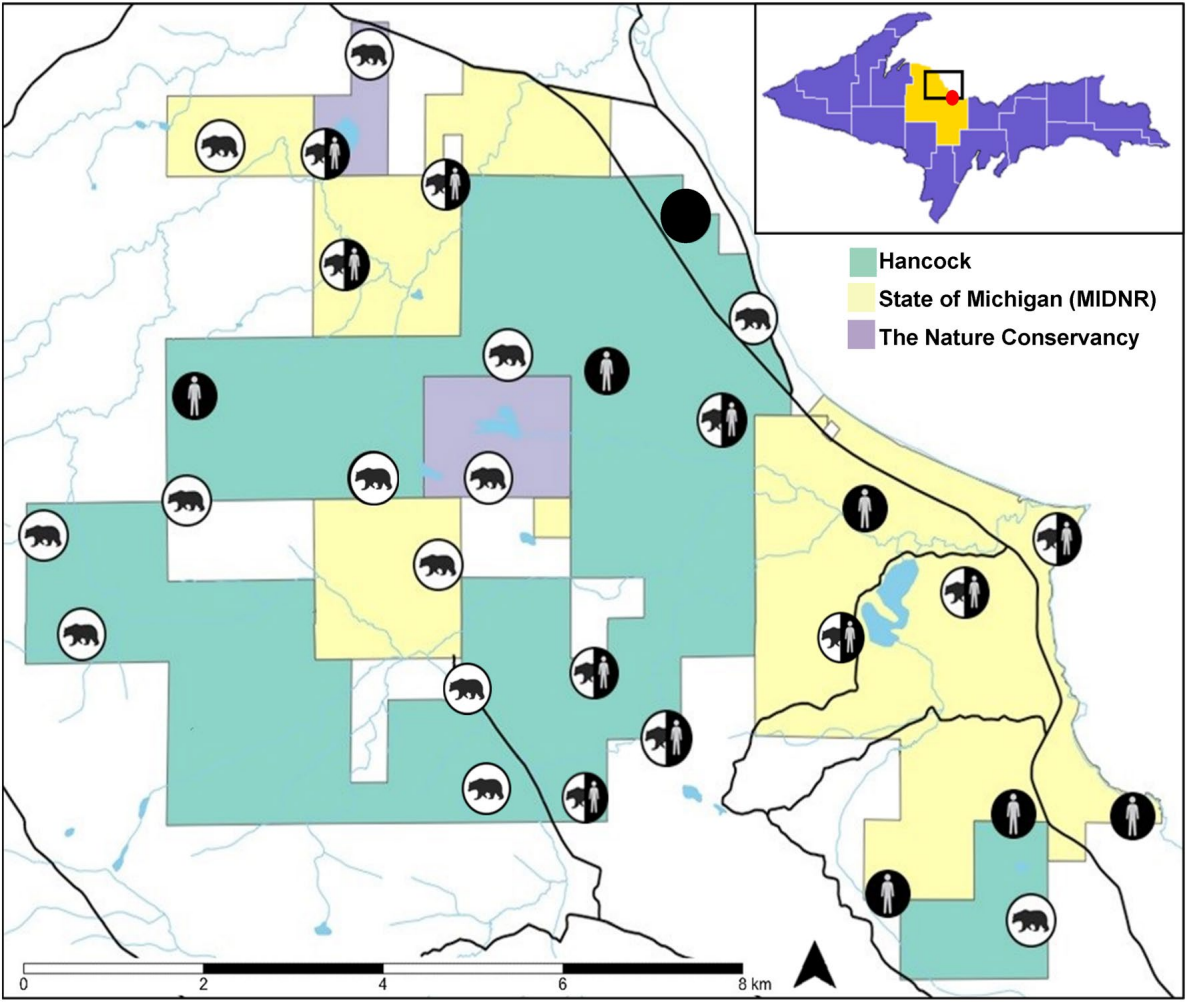
Study area map. Map of 60 km^2^ study area displaying current land management with circles indicating locations of camera traps and whether black bear, human, or both or none (i.e., solid black circle) were detected. Inset map of the Upper Peninsula of Michigan with Marquette County highlighted, study area indicated, and city of Marquette marked with red dot. Shapefiles and rasters processed using R (version 4.0.3; R Core Team 2020), R Studio (version 1.3.1093; R Core Team 2020), tidyverse (R, version 1.3.1), raster (R, version 3.5.2), sf (R, version 1.0.3).

We conducted our study in Marquette County, along the rural-wildland interface just north of the peninsula’s largest city, Marquette (46.5436° N, − 87.3954° W). The 60 km^2^ study area (Fig. 1) is bordered to the east by Lake Superior and covers an area that includes several popular outdoor recreation areas (e.g., Harlow Lake, Sugarloaf Mountain, North Country Trail) and commercial forest lands that experience considerable seasonal changes. Snow cover generally lasts from November to April with average temperatures reaching 23.6 °C in July and dropping to − 10.8 °C in January^38^. The area is under mixed management including Michigan DNR, The Nature Conservancy, and Hancock Timber Management Group. Land cover across the study area is diverse and includes coniferous, deciduous, and mixed forests, wetlands, occasional meadows, sand dunes, rocky outcrops, as well as an extensive Lake Superior shoreline.

### Camera trap surveys

We deployed 30 trail cameras (Primos Proof Generation 2) equipped with infrared flash across the urban-wildland interface between August 31 and September 8, 2019. To determine camera locations, we overlaid a 1 km^2^ grid across the study area and used a randomization method in the R package spatialEco^39^ to identify the center point of each of the 40 grid cells. While we only deployed 30 cameras, generating an additional 10 points allowed deployment flexibility when a grid cell was not accessible (e.g., private lands). Upon arriving at the approximate center of a grid cell, we searched for animal signs (e.g., animal trails, scat, etc.) within 100 m of the center point to identify locations that may increase the probability of capturing wildlife images. In some instances (n = 8), for example, because wildlife are known to use human trails and roads for travel^5,35^ cameras were placed near or in the direction of recreational dirt roads and old overgrown conservation roads that are occasionally utilized by vehicles. Cameras were strapped 0.5 m^40^ above the base of trees ± 30° of north to reduce direct sunlight^41^, and when possible, along linear features (e.g., river, trail, etc.) with no addition of bait or lure. Camera settings were chosen to increase the probability of capturing and accurately identifying fast-moving carnivores, thus cameras recorded multiple photographs per trigger, at a rate of 1 frame per second, re‐triggering immediately if the animal was still in view^5,41,42^.

We exchanged SD cards every 2–3 months, except during winter when many cameras were not accessible. In early November 2019, cameras were shifted to a height of 1 m above the ground in preparation of snowfall and any further height adjustments were made based on weather conditions and individual site conditions. As we were capturing images along a rural-wildland interface, images were sorted and all license plates and human faces were blurred to remove identifiable features. Following this procedure all images were organized as events and/or subjects based on a 5 s window (i.e., images were grouped if they were taken within 5 s of the previous image) and were uploaded to the Yooper Wildlife Watch project on Zooniverse (https://www.zooniverse.org/projects/bergq105/yooper-wildlife-watch), an online imagery platform where wildlife images can be managed, identified, and archived. Using this online platform allows for the global engagement of citizen scientists, and serves as an efficient way to quickly gather species information and image metadata for further data analysis. After completion of subject identification, which included 10 volunteers identifying the given animal and completing several additional tasks (i.e., number of individuals, adult or young, behavior, male or female) we pulled all subjects with at least 50% of volunteers identifying a black bear for further review. Following our expert review, we created independent wildlife observations or detections which were determined using a 30-min interval between sightings of the same species^43,44^. For consistency, this method was used for both black bears and human related detections, noting that on heavily traveled human trails there may have been different individual humans passing within 30 min of each other.

## Data analysis

### Analysis—temporal activity

Daily activity patterns of American black bears and humans were analyzed using the package “overlap”^45^ in RStudio version 1.3.1073^46^. Time was converted to radians to create kernel density estimation curves for (1) black bears before hibernation (i.e., all detections following camera deployment in September [bear hunting season] and before the final bear siting in the fall) and (2) black bears after hibernation (i.e., all detections following the first bear detection in the spring and before the month of September). The same was done for humans, domestic dogs, and vehicles using the timeframe established by the temporal span of black bear detections. Diurnal time boundaries were determined by calculating the average sunrise/sunset time from all black bear detections before and after hibernation. Overlap estimates were made for black bears before and after hibernation as well as for the different types of human activity using the overlap coefficient (Δ), which is scaled from 0 to 1, where (Δ = 1) signifies complete overlap^43,44,47^. We also investigated the effect of cub presence on black bear daily activity patterns to determine if their presence contributed to significant changes in activity, but given the smaller sample size of cub detections, we calculated this metric using combined data for both the before and after hibernation^48^. Although this will not show us differences between before and after hibernation, the presence of cubs in general may reveal critical information about black bear activity^28^. Further, activity level estimates were calculated using the package “activity”^49^ by fitting a flexible circular distribution to calculate the proportion of time a single species or group of individuals is active within a 24-h period^47^. After calculating activity level estimates, we used a Wald test to determine whether there was a significant difference between black bear activity level before and after hibernation, as well as between black bears and differing types of human activity including: (1) on-foot (i.e., hiking, snowshoeing), (2) non-motorized (i.e., cross-country skiing, biking), and (3) vehicles (i.e., normal, recreational, utility). Finally, we extracted temperature data from black bear images to plot monthly and hourly changes to determine if black bears display any threshold for activity based on temperature.

### Analysis—occupancy modelling

We created occupancy models to determine the: (a) probability of black bear detection at a site and (b) probability of a site being occupied by a black bear given several anthropogenic and environmental variables before and after hibernation. Binary detection histories (1 = detected, 0 = not detected) were created for black bears for the two time periods at each camera site. We accounted for imperfect detection by using weekly sampling occasions (Before: n = 14; After: n = 18), which reduced the number of observations where the count of detections is zero^20,50^. Camera trappers were not included when running occupancy models due to their presence at every site, which could impact the results. Data for environmental covariates and some human impact covariates, which consisted of large-scale human factors across the landscape, were extracted from geo-spatial layers available on the government Landfire database (https://www.landfire.gov/), USDA database (https://www.nass.usda.gov/), and SEDAC (https://sedac.ciesin.columbia.edu/) (Table 1). All data for human recreation covariates, which included fine-scale human presence across the landscape, were calculated from the collected camera dataset (Table 1).

**Table 1:** Summary of environmental and human covariates included in occupancy models based on values for each camera site before and after hibernation.

Model/covariate	Description	Max	Min	AIC value
**Human impact**				
*Human presence	Yes/no	–	–	480.70
*Human population density	Population density per 1 km sq	10.35	0	481.12
Road	Distance to nearest road (m)	1541.60	8.69	483.54
Land ownership	Hancock, Nat. Conservancy, MIDNR	–	–	484.57
Protected land	Yes/no	–	–	484.37
**Human recreation**				
*Human count	Total # of humans	589	0	481.84
*Human on-foot	Total # of humans on foot	14.97	460	482.20
*Human non-motorized	Total # of non-motorized rec	129	0	482.33
*Domestic dog presence	Yes/no	–	–	482.77
Recreation vehicle presence	Yes/no	–	–	485.14
*Passenger vehicle presence	Yes/no	–	–	483.09
Utility vehicle presence	Yes/no	–	–	484.80
Gun present	Yes/no	–	–	484.69
*Sum of human activity	# of dogs, humans, and vehicles	1034	0	482.84
**Environmental impact**				
Landcover type	Primary forest species (i.e., hemlock, etc.)	–	–	484.85
Water source	Nearest water source (m)	781	10	485.14
Elevation	Meters above sea level	435.02	183.06	484.85
Season	Before hibernation/after hibernation	–	–	483.37

(*) Indicates significant effect.

We checked for correlations between all continuous covariate pairs using the package corrplot^51^ with a threshold of 0.7 to indicate high correlation for eliminating covariates that encompass the same variation. Highly correlated covariates included human count, humans on-foot, domestic dogs, and humans on non-motorized recreation vehicles (i.e., bikes), which all had correlation values greater than 0.9. Covariates were grouped into three categories (1) human impact, (2) human recreation, and (3) environmental impact (Table 1). Single-species occupancy models were first run on a select group of covariates that were predicted to influence black bear detection probability. The covariate ‘season’ accounted for the differences before and after hibernation, as well as when the hunting season occurred, while the covariate ‘protected land’ takes into effect areas where hunting is permitted versus not permitted. We retained both ‘season’ and ‘protected land’ as covariates in our final occupancy models. Next, we ran each covariate on black bear occupancy probability and further used an information theoretic ranking (Akaike Information Criterion [AIC] values) from our single-species models to determine which covariates exhibited the strongest effects. After removing highly correlated covariates, we retained human presence, human population density, season, and protected land in our single final model to best predict black bear space use across the landscape (Table 1).

## Results

**Figure 2:**
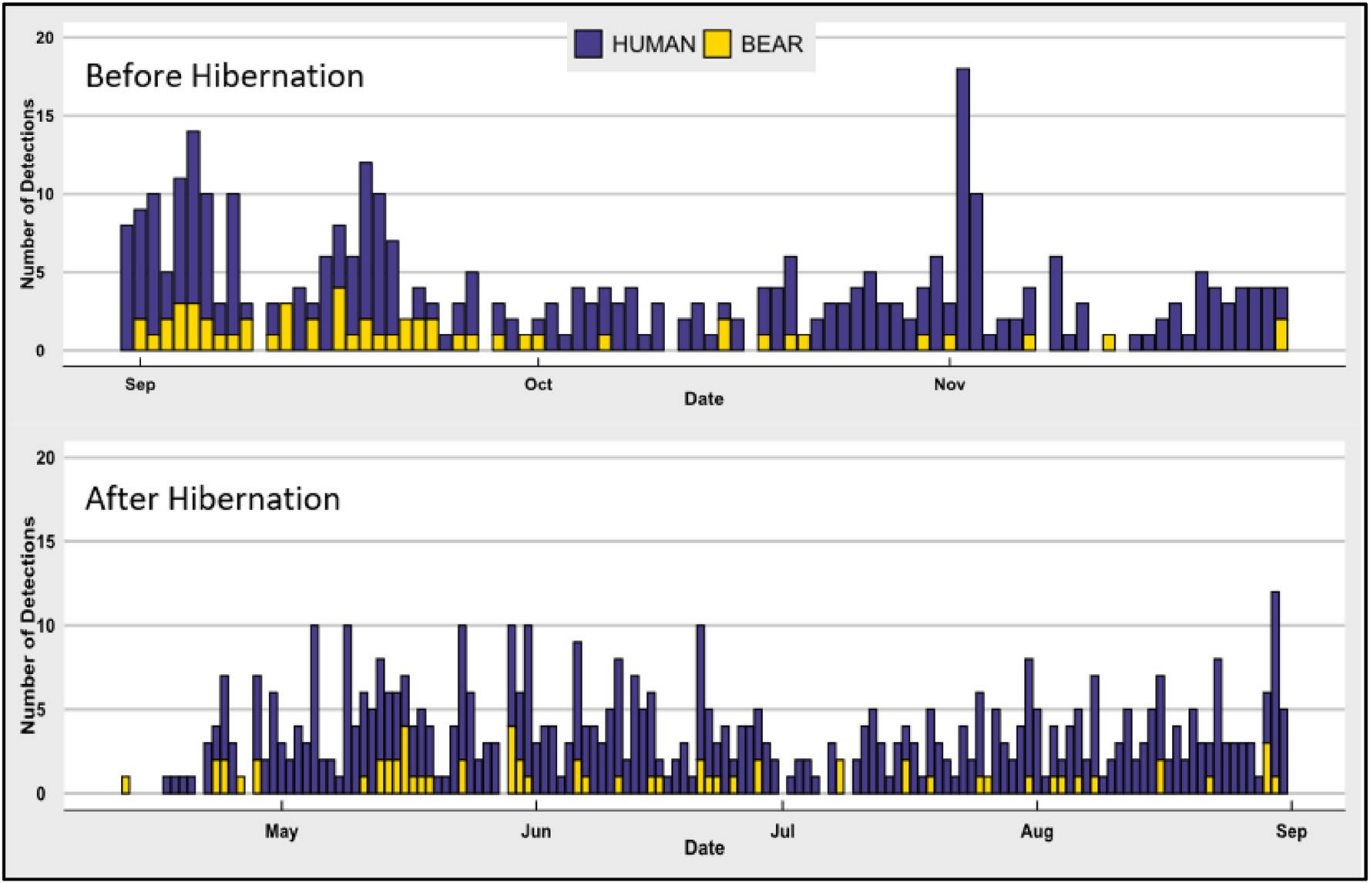
Summary of black bear and human detection history. Summary of when black bear and human detections were recorded throughout study period and further broken down into before hibernation (top), and after hibernation (bottom). The increase in human detection before hibernation in November represents deer hunting season.

Detections were recorded before hibernation (i.e., September 1st, 2019 to November 26th, 2019 and September 1st, 2020 to September 8th, 2020 [95 days]) and after hibernation (i.e., April 12th, 2020 to August 30th, 2020 [141 days]) for a total of 110 detections, 15 of which had a mother and cub, 2 of which had multiple adults, and 2 of which had only multiple cubs. Of the total detections, 46 were recorded before hibernation while 66 were recorded after hibernation (Fig. 2). Black bear detections were captured at 23 of the 30 camera sites with 42 independent black bear detections recorded at a single location. Upon further investigation, this location had a mother and cub frequently visiting the camera and likely denning nearby, yet other adults were still distinguishable when reviewing images collected at this site (Table 2). Approximately 48% of all black bear detections included direct physical interaction by the black bear with the camera.

**Table 2:** Summary of detections for American black bears, humans, domestic dogs, and vehicles before and after hibernation.

Species	# of locations	Before hibernation	After hibernation	Total
**Black bear**				
Adult/subadult	23	46	50	96
Cubs	10	13	16	29
**Human**				
On-foot	18	372	526	898
Non-motorized	1	34	129	163
With gun	3	13	0	13
**Dog**				
Collared	6	125	355	480
Not collard	7	49	75	124
**Vehicle**				
Recreational	5	20	23	43
Passenger	3	36	11	47
Utility	2	16	7	23

Human detections totaled 1191 with on-foot making up 898 of the detections and non-motorized accounting for 163 detections. There was an increase in human activity rate following hibernation in the spring by approximately 0.40 detections per day or about 9% (Before: 4.3/day; After: 4.7/day). Further, images were inspected for the presence of guns (i.e., hunters), which were recorded only before hibernation at three different sites and 13 independent detections (Table 2). Although the State of Michigan does allow bow and arrow, a crossbow or a firearm to be used for hunting^36^, all recorded hunters were seen carrying rifles.

Domestic dog detections totaled 611 with 480 collared dogs being captured at six different sites, 124 non-collared dogs being captured at seven sites, and seven detections where collars were indeterminate. Non-collared dogs made up approximately 20% of the total dog detections. Before hibernation, we recorded 1.8 dog detections per day and after hibernation 3.1 per day, thus there was approximately a 41% increase in the rate of dog detections in the spring (Table 2).

We recorded 113 independent detections of vehicles that were grouped into three categories: (1) recreational vehicle (i.e., four-wheeler, ATV, snowmobile), (2) passenger vehicle (i.e., average car or truck), and (3) utility vehicle (i.e., logging truck, dump truck, etc.). Vehicles were recorded at five different camera sites with a total of 43 recreational vehicles, 47 passenger vehicles, and 24 utility vehicles. Vehicles were recorded at a rate of 0.4/day after hibernation and a rate of 0.5/day before hibernation for an approximate 20% increase (Table 2).

### Temporal activity

**Figure 3:**
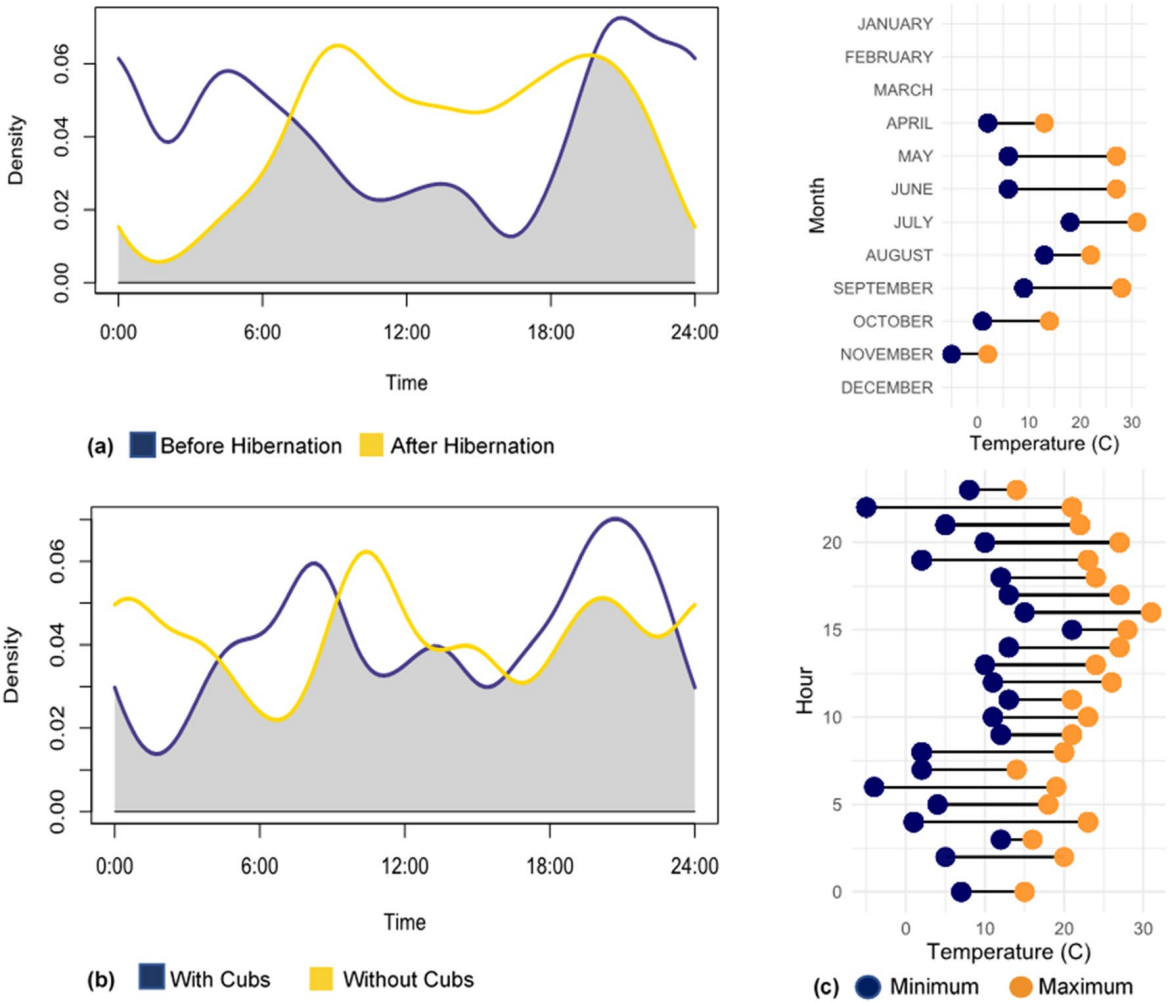
Temporal activity with relation to environmental temperature. (**a**) Black bear activity before and after hibernation. (**b**) Black bear activity with and without the presence of cubs. The gray area under the curves represents the overlap between the two activity patterns. (**c**) Minimum and maximum temperatures recorded for all black bear detections grouped by month and hour of the day.

Comparing temporal activity in American black bears before and after hibernation, we observed an increase in activity during diurnal hours by over 30% after hibernation (Before: 42.5%; After: 78.4%) with an overlap estimate of 0.661 (Fig. 3). A slight increase in black bear activity level was also observed after hibernation (Before: 0.574; After: 0.641) suggesting they were active for a larger proportion of the day though this difference was not statistically significant (p = 0.560). Variation in activity was also compared for bears with and without cubs using all detections recorded during the study period. We calculated an overlap estimate of 0.799 with bears exhibiting higher diurnal activity without cubs (With: 56.4%; Without: 59.6%) as well as a lower activity level (With: 0.669; Without: 0.594) that was not significantly different from bears with cubs (p = 0.598). We briefly investigated the relationship between black bear activity and temperature, in which we observed a consistent increase in the mean temperature from April (7.1 °C) to July (24.1 °C), and then a decrease moving into November 0.6 °C) as expected. Only two (~ 2%) black bear detections occurred at temperatures below freezing during the month of November.

Non-motorized human activity (i.e., biking) levels differed significantly before and after hibernation with an increase in the proportion of day they were occurring following black bear hibernation. Human activity consistently had higher overlap with black bear activity after hibernation, which could be a result of the overall increase in human activity observed in the spring. Among the different types of human activity, similar overlap estimates with bear activity were calculated that had exhibited no significant differences (Table 3).

**Table 3:** Human activity overlap (Δ) with black bears and activity level estimates before hibernation (BH) and after hibernation (AH).

Human activity	BH Δ	AH Δ	BH activity level	AH activity level
All recreation	0.325	0.621	0.361	0.340
On-foot	0.332	0.635	0.375	0.342
Non-motorized	0.229	0.582	0.209	0.339
Vehicles	0.317	0.573	0.328	0.292

### Occupancy modeling

**Figure 4:**
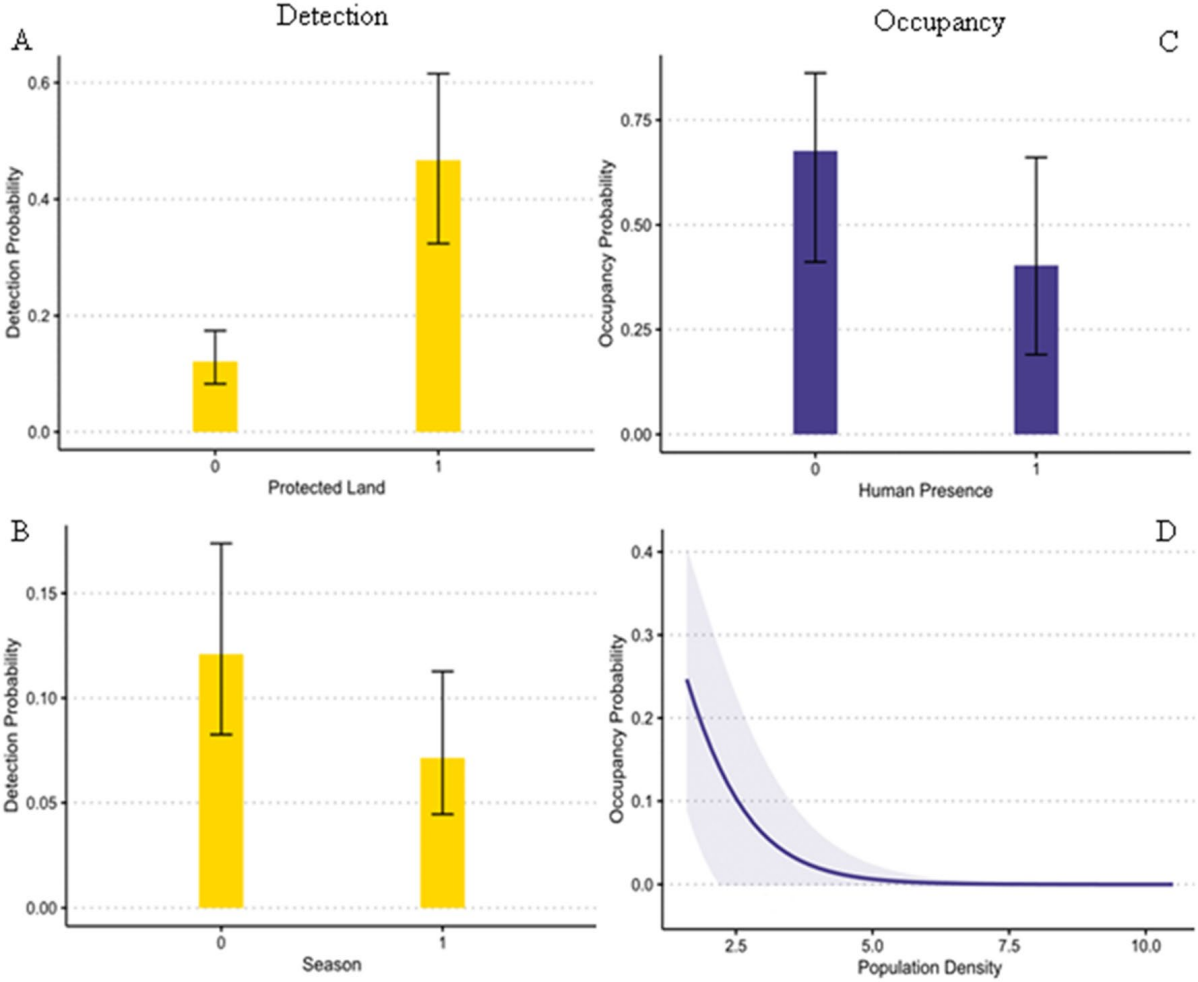
Top occupancy model results for detection and occupancy probability. Graphs in left column show the relationship between estimated detection probability and (**a**) protected land [0-unprotected, 1-protected], and (**b**) season [0-before hibernation, 1-after hibernation]. Graphs in right column show the relationship between estimated occupancy probability and (**c**) human presence [0-not present, 1-present], and (**d**) human population density. Shaded region represents the standard error.

Black bear occupancy was driven by human presence, human population density, and changes in seasonality that resulted in higher occupancy before hibernation (i.e., 18 sites occupied) than after hibernation (i.e., 14 sites occupied). Our final model showed a near significant negative effect caused by human presence (β = − 1.13 ± 0.63SE) and human population density (β = − 0.16 ± 0.61SE) (Fig. 4).

The probability of detecting a black bear in our final model was significantly associated with protected areas (β = 1.85 ± 0.33SE) and the season (β = − 0.67 ± 0.33SE) (Fig. 4). Season had a negative effect on black bear detection probability after hibernation, which could be related to a longer sampling window. Protected land areas greatly increased the probability of detecting black bears with a strong positive effect given that only three sites were located within protected boundaries.

## Discussion

Our research provides insights into the effects that human activity exerts on American black bear activity and occupancy across the urban-wildland interface, thus furthering our knowledge and ability to create better management practices in the region and in areas with recolonizing populations more broadly. Specifically, we investigated how different forms of human activity (i.e., human presence, domestic dogs, vehicles, etc.) and other environmental factors altered black bear occupancy before and after hibernation. Our findings showed that black bears are significantly affected by human presence across the landscape with variation in activity and occupancy observed before and after hibernation. However, because we found no clear differences in black bear activity based on the different types of human activity and recreation detected, our results suggest black bears might not differentiate among the different types of human activity and recreation occurring in the study area. Similar results have been observed in past studies showing that differences in bear activity patterns did not differ between sites with motorized or non-motorized recreation^31^. In addition, variability in responses to human features have been observed among bear species, such as black bears not displaying the same avoidance response to roads as grizzly bears (*Ursus arctos horribilis*)^31^.

We used kernel density estimation curves to analyze the temporal activity patterns of black bears to detect a consistent trend of increased overlap between bears and humans following hibernation, which we expect is partially due to a substantial rise in outdoor human recreation because of COVID-19 restrictions during our study^37^. This increase was observed for all types of recreation (> 38%) after hibernation, thus through further analyses, we concluded that the different types of recreation were highly correlated and human presence captured their effect in a single covariate. Given this, we observed no difference in black bear temporal activity due to variable human activity across the landscape. Bear activity was reduced during daytime hours prior to hibernation, which suggests that bears remained active throughout the day but shifted to peaks of activity in the twilight hours during and following the hunting season. Direct interactions with humans have also been found to alter temporal activity in the short term, with bears becoming less active for several days following the event^9^, thus limiting the time they spend foraging. We also investigated the effect of cub presence on black bear temporal activity, observing a small increase in diurnal activity when cubs were absent. The small sample size of detections with cubs present prevented us from analyzing before and after hibernation data separately, which may have limited our ability to detect an effect, though we do not expect black bear cub activity to vary substantially across seasons. We observed an increase in cub detections in the spring as expected, yet we would also expect the birthing of new cubs to increase the activity level and demand for resources by female black bears after hibernation forcing them to spend greater time searching for and consuming food. The risk of infanticide may also play a role in female black bears temporal activity, though previous research in the U.P. suggested that females do not change their space use patterns to avoid infanticide^52^. Before hibernation we would also expect to observe a high demand for resources that are becoming scarcer, forcing black bears to spend a larger time actively foraging to support the energy demands associated with the onset of hibernation^9^. As such, having a larger dataset might provide more evidence to support black bear activity driven by the energy demands of hyperphagia and the need for larger quantities of food and nutrients to support cubs following hibernation.

To explore the spatial scale of black bear activity, we used occupancy models and an array of human and environmental covariates. We found black bears to be negatively associated with human presence and human population density. Further, our results indicate that black bears are influenced strongly by human activity across the landscape, with little to no impact from the environmental factors that were measured (Table 1). Due to our small dataset for some human activity covariates, we suggest further investigation into specific types of recreation is necessary to fully understand black bear occurrence and behavior across the landscape. For example, our study only included a total of 13 detections where a hunter with a gun was clearly identified. We expected hunters to have a larger effect on black bear occupancy than the average human (i.e., hiker)^5^, but our sample size for this covariate was too small to provide meaningful insight. Moreover, we did observe a significant effect on black bear occupancy due to the season (i.e., before or after hibernation), yet a negative effect on occupancy was observed after hibernation, which does not correlate with hunting season. A possible explanation for this observation may be due to high resource needs before hibernation, driving bears to initially move greater distances in search of food, followed by reduction in activity as their metabolism and heart rate slows to supplement their necessary fat gain^53^. As food becomes more limited with the onset of winter black bears have been observed to travel consistent patterns while obtaining food^54^, which could lead to individuals maximizing the use of nutrient rich areas in the fall to avoid burning fat stores. Given this, the risk of finding food that allows for successful hibernation may outweigh the risk associated with human activity during the hunting season. Further, we found that protected areas had a strong positive association with black bear detection. Given only 3 sites (10%) were located within protected lands, no hunting zone or protected areas could also play a key role in determining how black bears use the landscape during hunting seasons. Finally, the increased amount of nocturnal activity exhibited by black bears in the fall could substantially increase their chance of avoiding hunters across the landscape due to legal hunting hours beginning 30 min before sunrise and ending 30 min after sunset in the state of Michigan^36^.

American black bears play important functional roles across variable ecosystems of North America, having critical life history traits that can be highly influenced by variation in human activity, weather, and resource availability across the landscape^27^. As such, ensuring black bear populations can meet their hibernation requirements should be a primary consideration for wildlife managers when considering hunting regulations for future bear harvest. The Michigan DNR has recorded data showing a 17% increase in the bear harvest from 2018 (est. 1521 bears) to 2019 (est. 1786 bears), with similar numbers in 2020^36^. We observed the highest number of black bear detections throughout the months of September and October, which takes place during hunting season and the time when black bears are preparing for hibernation, as well as an apparent shift in activity after hunting season despite the increase in human activity in the spring. Although the U.P. has had an active hunting season for many years, continuous monitoring of the population is necessary to ensure a stable population and will provide knowledge for wildlife managers in areas where hunting seasons are established in the future. Moreover, increasing our knowledge of how human hunting activity affects black bear temporal and spatial patterns is critical for understanding the impact that humans have on successful hyperphagia in black bears.

American black bears are a well-known and a frequently studied large carnivore that has been recolonizing and expanding their range across much of North America. Although highly impacted by human presence and human population density across the landscape, black bears have the capacity to coexist in human-impacted landscapes and even thrive in human-altered systems^10^. Investigating black bear temporal and spatial activity patterns in the U.P. where wildland is abundant yet easily accessible by humans provides substantial information to inform management practices associated with recolonizing populations across North America.

## Data Availability

The data that support the findings of this study are openly available in Zenodo at http://doi.org/10.5281/zenodo.5819191.
